# ASSESSMENT OF MAST IN EUROPEAN PATIENT-CENTERED TELEMEDICINE PILOTS

**DOI:** 10.1017/S0266462315000574

**Published:** 2015

**Authors:** Anne Granstrøm Ekeland, Astrid Grøttland

**Affiliations:** University Hospital of North Norwayanne.granstrom.ekeland@telemed.no; Norwegian center for integrated care and telemedicine

**Keywords:** HTA-approaches, MAST, Use and usefulness, European telemedicine pilots

## Abstract

**Objectives:** Model for ASsessment of Telemedicine Applications (MAST) is a health technology assessment (HTA) inspired framework for assessing the effectiveness and contribution to quality of telemedicine applications based on rigorous, scientific data. This study reports from a study of how it was used and perceived in twenty-one pilots of the European project RENEWING HEALTH (RH). The objectives of RH were to implement large-scale, real-life test beds for the validation and subsequent evaluation of innovative patient-centered telemedicine services. The study is a contribution to the appraisal of HTA methods.

**Methods:** A questionnaire was administered for project leaders of the pilots. It included questions about use and usefulness of MAST for (i) preceding considerations, (ii) evaluation of outcomes within seven domains, and (iii) considerations of transferability. Free text spaces allowed for proposals of improvement. The responses covered all pilots. A quantitative summary of use and a qualitative analysis of usefulness were performed.

**Results:** MAST was used and considered useful for pilot evaluations. Challenges included problems to scientifically determine alternative service options and outcome within the seven domains. Proposals for improvement included process studies and adding domains of technological usability, responsible innovation, health literacy, behavior change, caregiver perspectives and motivational issues of professionals.

**Conclusions:** MAST was used according to its structure. Its usefulness in patient centered pilots can be improved by adding new stakeholder groups. Interdependencies between scientific rigor, resources and timeliness should be addressed. Operational options for improvements include process studies, literature reviews and sequential mini-HTAs for identification of areas for more elaborate investigations.

Many reviews on the effectiveness of telemedicine conclude that high-quality studies of its outcomes are lacking, and some argue about the need for a general evaluation template for telemedicine (1–10). This was also the view of the European Commission (EC) which initiated the Methodologies for Assessing Telemedicine Applications (MethoTelemed) project in 2009. The development of the Model for ASsessment of Telemedicine (MAST) was one of the outcomes of this project (11). MAST's overall aim is to provide a structured framework for assessing the effectiveness and contribution to the quality of care of telemedicine applications. An underlying purpose is to develop a basis for investment decisions about applications or services.

MAST resembles a mini-health technology assessment (HTA), defined as “a form of checklist with several questions about the prerequisites for and consequences of using health technology” (12). Additionally, MAST recommends scientific methods and a multidisciplinary assessment of outcomes, comprising seven domains as worked out in the European network for Health Technology Assessment (EUnetHTA) (13). It is thus a comprehensive framework that combines HTA and mini-HTA approaches.

This article reports about an initial study on the use of MAST for assessing twenty-one telemedicine pilots in the EC project RENEWING HEALTH (RH) (14). A critical assessment of MAST's applicability in the telemedicine pilots was defined as one of the tasks within the RH project. The following objectives were described: (i) to examine whether the MAST framework works according to its aims and purposes; and (ii) to validate its applicability for different studies of telemedicine in pilots. The critical assessment was expected to potentially serve as a basis for a new version of MAST.

The initial study reported here was framed within these tasks in RH. This study reports and analyses the answers to the following questions: How was MAST used and perceived in the twenty-one pilots of RH? What were the recommendations for improvement of MAST?

The study applied a mixed methods approach and was conducted using an online questionnaire. Our main objective was to identify, conceptualize and analyze issues central to the discussion of MAST's further applicability and usefulness. Such conceptualizations should be germane to assessment frameworks in general; therefore, the study contributes to the appraisal of HTA methods.

## MAST FRAMEWORK

MAST defines the relevant assessment of telemedicine applications as a multidisciplinary process that summarizes and evaluates the outcomes of telemedicine, based on data in accordance with established scientific standards, and guidelines for data collection and outcome assessment. MAST does not recommend specific scientific methods because the methods and outcomes of each study depend on the objective, patient group, and intervention. MAST defines a three-level approach: (i) preceding considerations, (ii) assessments within seven domains, and (iii) considerations of transferability (11). Within the three levels, a thematic checklist is worked out as follows:

For the “preceding considerations,” users should assess the purpose of the telemedicine application under consideration, as well as relevant alternatives. Whether the assessment will address international, national, regional, or local issues should be decided, as well as the maturity of the technological application to be implemented.

The “multidisciplinary assessment” should be carried out within the following seven domains: the health problem and characteristic of the application, safety, clinical effectiveness, patient perspectives, economic aspects, organizational aspects, and sociocultural, ethical, and legal aspects.

“Transferability assessment” refers to considerations about the services’ potential for expansion. MAST recommends considerations of expansion across geographical and institutional borders, scalability, as well as generalizability according to statistical calculations.

### Objective of RENEWING HEALTH

The RH's objective was to implement pilots—large-scale, real-life test beds—for the validation and subsequent evaluation of innovative telemedicine services within a patient-centered approach.

The RH project comprised twenty-one different patient-centered, telemedicine pilots, clustered according to type of service and localized in nine regions across Europe. Table 1 gives an overview of the twenty-one pilots and their localization in clusters and regions (14).

**Table 1.** RENEWING HEALTH, Clusters and Regions

Type of service Pilot site	PATHOLOGY	VENETO	SYDDANMARK	NORRBOTTEN	NORTHERN NORWAY	CATALONIA	SOUTH KARELIA	THESSALY	CARINTHIA	BERLIN
Cluster 1 Medium-term health coaching and life-long monitoring	**DIABETES**			**X**	**X**		**X**		**X**	
Cluster 2Life-long monitoring		**X**						**X**		**X**
Cluster 3Ulcer monitoring			**X**							
Cluster 4Short term follow-up after hospital discharge	**COPD**		**X**			**X**		**X**		
Cluster 5Life-long monitoring		**X**							**X**	**X**
Cluster 6Medium-term health coaching and life-long monitoring	**CVD diseases**			**X**			**X**			
Cluster 7Remote monitoring of Congestive Heart Failure		**X**						**X**		
Cluster 8Remote monitoring of implantable cardiac devices (ICD & PM)		**X**								
Cluster 9Medium-term health coaching and life-long monitoring, with high blood pressure				**X**						
Cluster 10Life-long monitoring of frail patients with chronic diseases		**X**								

The achievements of the RH objective were planned to be monitored and documented by using MAST. Different scientific studies, including PhD projects, were also planned along with the pilots. These will not be addressed here.

This study is structured according to MAST's three levels: preceding considerations, assessment within seven domains, and transferability. MAST's aims to induce and take into consideration assessments based on scientific standards and guidelines with the underlying purpose of developing a basis for investment decisions is also addressed. The identification of issues for discussion is accounted for in the methods section.

### METHODS, DATA, AND ANALYTICAL PERSPECTIVES

The RH partners were offered a MAST seminar, introducing the framework and several materials: a MAST manual, a database, and guidelines for data coding and analysis and reporting of results. The initial study reported here was initiated in October 2013. The assessment was carried out through an online questionnaire (Supplementary Questionnaire) (14).

In the questionnaire, we first asked for a description of the pilot(s) on which the respondents reported. The intention was to assess whether all pilots were covered. We then provided questions in accordance with the MAST structure – whether the pilots had addressed the three levels and the specified topics within them. The questionnaire also contained options for free text comments within all three levels, where any use-related issues could be raised.

The questionnaire also included questions about a tool for reporting the MAST assessments from the pilots. Additionally, one section at the end of the questionnaire was designed for any comments and proposals for improvements.

The questionnaire was designed by using Google docs. The responses were collected electronically, and statistical options facilitated the representation of numerical data through different diagrams. All free text responses were registered sequentially according to their respective serial numbers on the questionnaire and dates when the responses were received. This allowed for successive reading and analysis of all responses to each free text question.

### Selection of Participants

The pragmatic selection of the study participants was intended to obtain data from all pilots and to approach the project leaders of each pilot. However, some adjustments were made. The country project manager/coordinator was responsible for coordinating the answers, but in two cases, different pilots were conducted within the same region. For this reason, project leaders and coordinators were given guidelines, informing them that for pilots within the country that were similar in terms of equipment, procedures, and data collection, one joint answer would be sufficient. They were also encouraged to initiate a discussion among the participants of the different pilots to find out if the responses were similar. In case the pilots were completely different and probably also involved different people who had used MAST, separate answers were expected.

### Data and Analytical Perspectives

The topics of interest were use, usefulness, perceptions of usefulness, and areas for possible improvement of the framework. The discussion section is based on a combination of factual results on the use of different parts of MAST and the number of users, as well as the participants’ comments. This way of combining data reflects a mixed-methods approach (15). By interpretively linking the qualitative data from the comments, the facts of which MAST elements that had been used and the numerical data on the users, we identified thematic areas for discussion to answer the qualitative questions.

This approach is linked with philosophical pragmatism, which views knowledge as being both constructed and based on the reality of the world we experience and live in (16). The knowledge produced in this study is based on the reality of use, as well as the participants’ constructions of meaning as expressed in the topics of their concerns about usefulness and proposals for improvement. The authors construct knowledge in this study by selecting certain topics from the data for analysis, based on the research questions. The study does not address the evidence for MAST's usefulness and performance; therefore, statistical analysis of answers is not provided.

The selection of themes for analysis is briefly introduced below. Concerns that reimbursement highly affected use were expressed along with a proposal to add production of knowledge about how reimbursement could be changed to the preceding considerations. Two interrelated issues are made topical concerning usefulness and improvement.

The first one concerns basic assumptions about forces causing change. A basic assumption embedded in MAST is that telemedicine interventions cause or produce outcomes in different domains. By pointing to reimbursement issues as hampering use, economic issues are introduced as crucial for outcomes. A classical discussion about technological determinism versus economic determinants, for instance, is thereby introduced (17;18). Such issues are highly debated both in health care and for institutional change in general (19;20).

How might such underlying assumptions affect MAST's usefulness and be involved in improvements of MAST? These questions also relate to the second discussion theme, based upon the proposal of adding an assessment of reimbursement with the purpose to change it. This proposal suggests an action-oriented research approach (21). Process and action oriented approaches are different from studies of the effects or outcomes of telemedicine as embedded in MAST, which presuppose technological determinism (10).

Furthermore, the users expressed concerns about access to evidence-based scientific results and that the framework did not consider local circumstances. Hence, discussions about scientific rigor and the balance between too much complexity and local relevance are pertinent.

To sum up, the following issues are selected for analysis concerning MAST's usefulness and proposals for improvement: (i) the framework's position on determinants or agency, including considerations of the level of analysis; (ii) the framework's position on process and action-oriented approaches; (iii) interdependencies among scientific rigor, resources and timeliness; and (iv) the framework's position on the balance between complexity and local relevance. The conclusion points to the framework's possible areas for improvement.

## RESULTS

The distribution of the questionnaires and guidelines to project leaders resulted in eleven responses covering all twenty-one pilots, clusters, and regions, indicating a 100 percent response rate. The responses came from northern Norway (Diabetes Mellitus [DM]), central Greece (DM, chronic heart failure [CHF] and chronic obstructive pulmonary disease [COPD]), Norrbotten region, Sweden (DM and CHF), Veneto region, Italy (DM, COPD, CHF, and chronic diseases), Veneto region, Italy (implantable cardiac devices), Catalonia, Spain (COPD), southern Denmark (COPD and DM), southern Denmark (diabetic foot ulcers), Carinthia, Austria (DM), south Karelia, Finland (DM and CHF), and Berlin, Germany (DM and COPD).

### Preceding Considerations

Preceding considerations suggest that the degree of development of the telemedicine intervention and relevant alternatives should be considered before the outcomes are assessed in the second step. Table 2 presents the number of users who had assessed the different issues.

**Table 2.** Preceding Considerations

Topic	No. of users
Considerations of maturity (e.g., based on pilot studies with a few patients)	11
Considerations of relevant alternatives	6
Considerations of international, national, regional, or local level of assessment	7
Consideration of legislation	9
Consideration of reimbursement	8
Consideration of the number of patients	9
Other	0

### Users’ Perceptions of Preceding Considerations

Ethical challenges were described for the consideration of relevant alternatives in the preceding phase. One respondent commented on expenditure reduction, which might override quality considerations for vulnerable patient groups:

“Patient maturity to use this kind of information and communication technology (ICT) applications was important, because the patients with these diagnoses are mostly elderly people. It was, therefore, also important to adapt the user interaction to their preferences and capabilities. This was performed in the preproject MyHealth@Age. The only alternative found was manual health coaching. But that was too expensive to be used [on a] large scale. Therefore, it was natural to perform the RH trial to get evidence that this kind of more resource-efficient method generates similar advantages as manual health coaching but more economically”.

The same user also commented on an economic consideration that had been brought forward during the preceding considerations and launched a possible improvement of MAST in this respect:

“Reimbursement was brought up as an important aspect to make the method (referring to the service in the trial) possible to launch [on a] large scale. But it was not possible to develop new reimbursement regulations for the trial”.

The respondent pointed to action-oriented process studies:

“Instead, the trial should generate information and knowledge on how the reimbursement structure should be developed to be appropriate for large-scale deployment”.

Another respondent described the keywords provided as relevant to gain a better understanding of the problem and to take into account important lessons and guidelines. Preceding considerations of the effectiveness and potential outreach of the intervention, as well as legal and reimbursement issues were prominent. These thematic areas within the preceding considerations were reported to “help design the service to be tested adequately, to specify the processes with physician and nursing services, and to determine the relevance and timeliness of the assessment to establish an enduring project”.

One respondent also pointed to the need for assessing technological usability and interoperability issues as part of the prepilot phase. Another one underscored that MAST was a tool for evaluating mature technologies; therefore, these preceding considerations were crucial. Regulatory legislation and reimbursement conditions were reported as important to describe at this stage because they were perceived as potential barriers to further implementation and scaling up.

To improve MAST, the inclusion of questions aimed at generating knowledge to be used for changing reimbursement structures was proposed.

### Assessments within All Seven Domains

Ten of the eleven respondents had considered all seven domains. All respondents had considered four of the seven domains, including health problems, clinical effectiveness, patient perspectives, and economic issues. This suggests that one respondent had not considered safety, organizational aspects, and sociocultural, ethical, and legal aspects.

### Users’ Perceptions of the Seven Domains

Eight respondents (67 percent) replied positively to the question: Were the seven MAST domains covering all potential aspects of the quality of care of your service(s)? Four answered partly, including one who also responded yes. The following four challenges were described in the free text space, one by each of these four respondents:

• The number and scope of domains were not regarded as the main challenge, but rather the difficulty to obtain scientifically rigorous knowledge within them.
• The caregiver's role was regarded as important, especially for dependent patients with chronic conditions. The necessity to assess this group's views and preferences separately and in addition to patient perspectives was pointed out.
• Health professionals differed in their perceptions of telemedicine's usefulness, suggesting the need to assess specifically their predisposition to use technology in routine clinical practice. Using a questionnaire based on the Technology Acceptance Model (TAM) was proposed. This model is briefly described in the discussion section.
• The technologies’ ease of use, considered crucial for patient motivation, was proposed as an additional domain.

### Transferability

Eight of the respondents performed a generalizability assessment; for three of them, this was the only transferability assessment that they did. Five of those eight respondents also considered cross-border and scalability assessments. Scalability alone was considered by one respondent, while cross-border and generalizability assessments were considered by two.

### Users’ Perceptions of Transferability Assessments

Three respondents commented on their answers. One stated that, in addition to scalability and generalizability, applicability to other patient groups was important to consider. Another respondent noted that this part of MAST was not adequately explained in its manual. A third one reported that the results of their team's pilot trial would be used by their Ministry of Health to determine the future policy on telemedicine deployment in their country. Using MAST helped them produce their results.

### MAST's Purposes: To Induce Assessments Based on Scientific Standards and Guidelines

Nine of the respondents reported on MAST's usefulness in providing a multidisciplinary basis for investment decisions about their services. Two responded “not certain,” explaining their views as follows:

“The first pilot had not run long enough, and it was too early to decide”. The second one referred to limitations “because of local circumstances, which influenced the perception(s) of both patients and staff”.

Does using MAST provide sufficient basis for evidence about investment decisions in telemedicine? Eight responded yes to the question, two were “not certain”, while one did not respond. Five respondents provided additional comments—the two who were not certain and three who answered yes. The comments included doubts because “differences in circumstances [between] the randomized controlled study (RCT) performed in the pilot [and] the real situations of use were anticipated, especially concerning costs”. In one case, the sample was considered too small to obtain evidence.

Concerning MAST guidelines’ applicability and relevance to the analysis and reporting of the results within the seven domains, nine of the regions reported improvement of the scientific quality of the analysis, while three did not find that the guidelines influenced the quality. One region responded both yes and no. This region commented that the guidelines gave a clear toolkit for the analysis to all partners but did not provide enough room (time) to dig deep enough into scientific results.

### Additional Suggested Improvements for MAST

One respondent wanted more emphasis on technical assessment, responsible innovation, attitudes, behavioral change and health literacy as important for the development of services. Another one underscored the importance of assessing the caregiver's role, especially when the patient was older with chronic conditions. A short version of MAST was proposed as a helpful tool, but the respondent did not elaborate in detail. One of the pilot sites considered it important to examine how the results of an assessment based on the MAST could be described and presented in a simple way for decision makers.

## DISCUSSION

The purposes of the initial study in twenty-one European pilots were to examine MAST's use, the perceptions of its usefulness, and proposals for improvements. The overall impressions were that MAST was used to assess: (i) preceding considerations, assessment within seven domains, and transferability; (ii) whether services were based on scientific standards and guidelines for developing a basis for investment decisions; and (iii) applicability and relevance to patient-centered pilots

The majority of users had done assessments within all the thematic areas provided for each level. For preceding considerations, issues of maturity, legislation, and reimbursement were salient. Outcome from all seven domains had been considered by ten of the eleven respondents and eight had performed a generalizability assessment. As such, MAST served as a practical tool.

### Perceptions of MAST's Usefulness, Challenges, and Proposals for Improvement

Within the preceding considerations, two challenges were prominent. The first one was reimbursement for well-functioning services and for extending these, leading to proposals that the framework should include a section to generate knowledge useful for changing reimbursement structures. Second, problems in obtaining scientific and rigorous knowledge on maturity and relevant alternatives were put forth. Concerning the purpose to induce assessments based on scientific standards and guidelines, problems in obtaining scientific evidence were also described within the seven domains. Concerning applicability and relevance to patient-centered pilots, new domains and stakeholders were suggested for the improvement of the framework's local relevance, as follows: technological usability, responsible innovation, health literacy, behavioral change, caregiver perspectives, and motivational issues of professionals.

### Reimbursement; Determinants, Agency, Level of Analysis, and Usefulness of MAST

Technological determinism stands in opposition to a theory of social construction of technology, which holds that both the path of innovation and the consequences of technology for humans are strongly shaped by society itself, through the influence of culture, politics or economic arrangements (18). In the study, economic arrangements were read to outplay the agency of technologies by hampering their expansion, as the reimbursement structure did not support large-scale deployment of certain services. The continuation of well-functioning services was also regarded as hampered by reimbursement structures.

One of the proposals for improving MAST was to add a domain for addressing professionals’ attitudes toward technology. Using the TAM model was suggested. This model replaces certain attitude measures developed within the theory of reasoned action (TRA) (22) with two technology acceptance measures – ease of use and usefulness. The TRA has strong behavioral elements, assuming that when someone forms an intention to act, he or she will be free to act without limitation, that is, agency and determinism are ascribed to individual behavior (23). The level of analysis may refer to the micro or macro level, a model's logical structure from basic assumptions to operational aspects, the temporal aspect, static versus dynamic, and the logical relationships between causes and outcomes (24).

One of the regions expressed concern about ethical issues of patient maturity when the target groups were elderly people with chronic illnesses. The region was assessing new services to save money even if the existing ones—personal health coaching—were good. These concerns could be addressed at the individual and operational levels within MAST's ethical domain. Additionally, the concerns point to causes within macrostructures that affect ethics, namely policy decisions on resources for elderly and chronically ill people. Considerations about how MAST should include attention to macroeconomic principles and their impacts on the implementation of telemedicine could be relevant to its usefulness.

Determinants or causal agency reflects assumptions about the nature of causality—whether external forces such as technological development or economic resources cause change, whether people purposefully act and accomplish intended objectives or whether changes emerge unpredictably from the interactions of people, resources and events. In MAST, the underlying assumptions seem to be that technologies at micro and macro levels cause outcomes. Technological determinism is prominent.

This is an insufficient assumption, judging from the responses. Preceding considerations were described as crucial, and especially legislation and reimbursement (which are macrostructural issues), to assess transferability and scalability before new technology would be implemented. Assumptions about agency and determinants and the level of analysis should be clarified to improve usefulness in future versions of MAST.

### Framework's Position on Process and Action-Oriented Approaches

Based on the ethical concerns that useful services might be replaced by telemedicine because it was less expensive, assessments to generate knowledge on how to develop new reimbursement models were proposed. These suggestions indicate that MAST should be useful not only for assessing outcome of telemedicine within given economic preconditions, but also for generating knowledge useful for improving such conditions. In that case, preceding considerations would include focus on a process aimed at changing economic conditions to arrange for ethical interventions. The suggestion, therefore, points to a formative assessment methodology, including forms of action research used for coupling research and action. According to Baskerville and Myers (25): “Action research aims to solve current practical problems while expanding scientific knowledge”. By including an action research component, MAST could be a dynamic tool for addressing power issues and contribute to changing conditions for certain outcomes.

### Interdependencies among Scientific Rigor, Timeliness, and Resources

Challenges to “dig deep enough” within preceding considerations and the seven domains were reported. The quality of the information in cases examined by mini-HTAs has been assessed by Kidholm et al. (12). They contend that the quality of assessments in many cases is insufficient, pointing to a strong need for quality assurance of mini-HTAs to improve the accuracy of information without harming the timeliness and limited use of resources in producing the reports. As both the preceding considerations and the seven domains involve major fields of research where scientific knowledge is increasingly complex, rigorous, and under development, this is an ongoing topical challenge. Using the MAST framework for quick pilot assessments to provide a basis for decisions, therefore, involves uncertainty and the risk of not being rigorous enough. Balancing scientific rigor, timeliness, and resources in changing environments takes knowledge, skills, and creativity; therefore, the MAST framework could benefit from including a section that would address such interdependencies.

### Balancing Unwieldy Complexity and Local Relevance

One of the proposals for improving MAST was to expand the domains and include new stakeholder groups, such as family caregivers. New domains and stakeholders for improving pilot assessments add not only practical relevance but also complexity and scope. Added complexity will likely increase tensions among practical relevance, scientific rigor, and timeliness.

On one hand, an ongoing process for setting technical standards for health care ICT has been considered critical, but needs to include the interests of all relevant stakeholders. Christensen and Remler (26) argue that such processes must be careful (slow), flexible and allow for as much diversity as possible.

Referring also to the previous discussion on agency, the success of patient-centered innovations has on the other hand been described as dependent not on the quality of technology per se nor on the evident need but on the overall coherence of a complex sociotechnical system (27). The complex sociotechnical system addressed in MAST involves reimbursement, legal regulations, technology, and the needs of different stakeholders. A section to address their interdependencies and the speed of change could also strengthen the framework's local relevance.

To address the complexity embedded in the users’ comments and proposals for improvement, operational responses in MAST should include performance of rigorous literature reviews within the preceding considerations. By combining review results with use of sequential short assessments according to the seven domains for the empirical cases, areas where more detailed assessment is required to inform decisions could be identified. Using process assessments for on-going adjustments of services as they develop is also an operational response to users’ concern. However, it is also important to notice that too many additions to MAST might well change it from a framework for relatively rapid assessments to something more elaborate which could be more difficult for potential users to complete.

## CONCLUSIONS

In the twenty-one pilots, MAST was used according to its aims to include preceding considerations, assessments within seven domains and assessments of transferability. The majority of users had done assessments within the thematic areas provided for each level. For preceding considerations, issues of maturity, legislation, and reimbursement were salient. All seven domains had been considered by ten of the eleven respondents and eight had performed a generalizability assessment. MAST's purpose to base assessments on scientific standards and guidelines was not fully accomplished, because such knowledge was unavailable for specific subject areas. The framework also displayed weaknesses in functioning as a basis for investment decisions. MAST's relevance as an assessment framework used in patient-centered pilots could be strengthened by more explicitly stating its position on the following issues: causal agency and determinants; process and action oriented approaches; levels of analysis and tensions among scientific rigor, resources, and timeliness; and ways that interdependencies of domains and stakeholders could be included in investigations without increasing the complexity beyond practical usefulness. Operational options might be scientific rigorous literature reviews combined with sequential assessments for identification of areas for more elaborate investigations. Action oriented process studies of complex conditions should also be added.

## Supplementary material

For supplementary material accompanying this paper visit http://dx.doi.org/10.1017/S0266462315000574. click here to view supplementary material

## CONFLICTS OF INTEREST

The authors declare no conflict of interest.
