# Supplementary material for: ASSESSMENT OF MAST IN EUROPEAN PATIENT-CENTERED TELEMEDICINE PILOTS
Source: Int J Technol Assess Health Care. 2015;31(5):304–11. doi: 10.1017/S0266462315000574 (PMC4762236; doi:10.1017/S0266462315000574)
Supplement: Supplementary file 1 [file S0266462315000574sup001.docx]

Supplementary questionnaire 1

Critical Assessment of MAST

1. What is the name of the pilot or pilots you are reporting from? *

…………………………….

2. Please provide an overall description of the intervention in your regional pilot(s) *

A short description of technology and services, no more than 100 words

………………………………………………

3. MAST includes as a first step that preceding considerations are made before a multidisciplinary assessment is made in the second step. Did you carry out preceding considerations of your local telemedicine application before RH was initiated? *

You may tick as many boxes as you like

Check all that apply.

|  | Consideration of maturity, e.g. based on pilot studies with a few patients? |
| --- | --- |
|  | Considerations of relevant alternatives? |
|  | Consideration of international, national, regional or local level of assessment? |
|  | Consideration of legislation? |
|  | Consideration of reimbursement? |
|  | Consideration of the number of patients? |
|  | Other: |

4. Why were the preceding considerations relevant or not relevant for your pilot? *

………………..

5. Were the 7 MAST domains covering all potential aspects of the quality of care of your service(s)? *

Check all that apply.

|  | Yes |
| --- | --- |
|  | No |
|  | Partly |

6. In case you ticked no or partly in the checkboxes, please indicate alternatives:

What would you add or delete from the structure to improve assessment of quality of care?

7. Did use of MAST provide a multidisciplinary basis for decision on investment in telemedicine? *

If you want to add a comment, please use the text field after the "other" box

Check all that apply.

|  | Yes |
| --- | --- |
|  | No |
|  | Not certain |
|  | Other: |

8. Will your use of MAST provide a sufficient evidence based basis for decision on investment in telemedicine? *

If you want to add a comment, please use the text field after the "other" box

Check all that apply.

|  | Yes |
| --- | --- |
|  | No |
|  | Not certain |
|  | Other: |

9. Did your use of MAST include assessment within all 7 domains? *

Please tick the domains which you assessed

Check all that apply.

|  | Health problem and characteristics of the application |
| --- | --- |
|  | Safety |
|  | Clinical effectiveness |
|  | Patient perspectives |
|  | Economic aspects |
|  | Organisational aspects |
|  | Socio-cultural, ethical and legal aspects |
|  | Other: |

10. MAST includes as a third step an assessment of the transferability of the results from your local pilot. Have or will you do a transferability assessment? *

Please tick as many alternatives as you need and write your comments in the "other" field

Check all that apply.

|  | Cross-border |
| --- | --- |
|  | Scalability |
|  | Generalisability |
|  | Other: |

11. As a part of Renewing Health a guideline for analysis and reporting of your results has been produced. Has the guideline had impact of your analysis of the results within the MAST domains? *

Mark only one.

|  | Yes, this has improved the scientific quality of the analysis and results |
| --- | --- |
|  | No, this did not have any impact |
|  | Yes, this has reduced the scientific quality of the analysis and results |
|  | Other: |

12. Do you have any suggestions for improvement of MAST which is not addressed in the questionnaire?

Please write your suggestion or any other comments

13. Do you have any comments on the relevance of using MAST for your pilot which is not addressed in the questionnaire? *

Please write your comments
